# Early Detection of Academic Performance During Primary Education Using the Spanish Primary School Aptitude Test (AEI) Battery

**DOI:** 10.3389/fpsyg.2020.630803

**Published:** 2021-01-21

**Authors:** Ignasi Navarro-Soria, José Daniel Álvarez-Teruel, Lucía Granados-Alós, Rocío Lavigne-Cerván

**Affiliations:** ^1^Department of Developmental and Educational Psychology, University of Alicante, Alicante, Spain; ^2^Faculty of Education, Valencian International University, Valencia, Spain; ^3^Department of Developmental and Educational Psychology, University of Malaga, Málaga, Spain

**Keywords:** cognitive skills, mathematics, language, learning difficulties, primary education

## Abstract

The aim of this study was to assess the predictive capacity of some of the most relevant cognitive skills pertaining to the academic field as measured by the Spanish Primary School Aptitude Test Battery. This psychometric tool was applied to all students who were enrolled in the final year of Early Childhood Education (631 students) in the public schools of the province of Alicante (in the South-East of Spain) and a follow-up of their academic progress was carried out when they completed Primary Education (6 school years). The results obtained show that medium-high and high scores in Verbal Aptitude and Numerical Aptitude tests in Early Childhood Education (5 years of age), can predict academic success at the end of Primary Education (12 years of age) in instrumental subjects such as: (1) Language (Verbal Aptitude Odds Ratio = 1.39 and Numerical Aptitude Odds Ratio = 1.39) and (2) Mathematics (Verbal Aptitude Odds Ratio = 1.47 and Numerical Aptitude Odds Ratio = 1.52). We have determined the importance of developing pedagogical programs that stimulate the development of these skills during Early Childhood Education, while implementing support strategies during Primary Education, for those students who present underdeveloped aptitudes in these areas. In this way, school difficulties would be prevented in the instrumental subjects that provide access to other academic areas.

## Introduction

The starting point for the present study was a certainty in the knowledge that academic difficulties do not suddenly appear without warning, but that they develop throughout the early stages of the teaching-learning process (Kern and Friedman, 2009; Garon-Carrier et al., 2018). From a child’s first steps, differences in the level of progress of his/her different capacities begin to occur, and in most cases individuals compensate possible deficiencies with motivation and a positive attitude toward the task to be learned. However, a large number of situations are observed whereby these difficulties prevent children from acquiring, in a reasonable period of time initial knowledge which is fundamental in the construction of further and more extended knowledge (Garon-Carrier et al., 2016).

In the body of literature on the field of educational research, academic performance is presented as worthy of constant concern. Over the years, it seems that there has been a change in the focus of these studies and currently the emphasis is on finding causal relationships between academic performance and different variables that may be the subject of research on intervention programs, such as math skills, language skills, spatial orientation, memory, or cognitive and psychomotor maturity to develop reading and writing skills (Risso et al., 2010; Alloway and Passolunghi, 2011; Phillipson and Phillipson, 2012; Cheng and Mix, 2014; Bonti and Tzouriadou, 2015; Geary and VanMarie, 2016; Hill et al., 2016; Park et al., 2016; Pitchford et al., 2016; Serpell and Esposito, 2016; Cornu et al., 2017). Therefore, it is evident that a special effort must be made in identifying and defining the variables that can be manipulated, controlled or modified in order to improve academic performance (Rohde and Thompson, 2007; Gay, 2010; Carlson et al., 2013; Núñez et al., 2014; Toll and Van Luit, 2014; Cerda et al., 2015; Puerta, 2015; Cassidy et al., 2016). At the same time, to our knowledge, in the body of specialized literature the definition of academic performance is understood to be the learning out come in the student and generated by the pedagogical intervention of the teacher, keeping in mind that this performance cannot be the product of a single aptitude, but is the result of the symbiosis of a sum of elements that act upon, and emanate from, the person who learns (Córdoba et al., 2011; Miñano and Castejón, 2011; Moliner et al., 2012; García-Fernández et al., 2013; Grivins, 2013; Meneses-Botina et al., 2013; Duckworth and Yeager, 2015).

On the other hand, low academic performance affects a large number of students and can pose a particular problem in each case. Low academic performance should not be understood as being associated to learning disorders, but rather associated to slight difficulties in the ordinary acquisition of curricular contents. A high percentage of students who show underdevelopment in their performance during Primary Education corresponds with children who have not presented obvious problems in their school development during the previous cycles (Pérez et al., 2005; Robles and Vázquez, 2014; Navarro-Soria, 2016). The increase in curriculum demands as the student advances, and the detection and late initiation of classroom reinforcement systems, aggravates the difficulties and increases the differences in levels of development in relation to other students in the classroom. Although these differences become more noticeable from around the mid-point of Primary Education, the problem arises during Early Childhood Education and can be noticed during the first stage of Primary Education, when the students face complex learning processes, such as the acquisition of reading and writing, numerical calculation or problems solving (Prior et al., 2011; Ye et al., 2016).

Therefore, it would seem evident that there is a need to detect whichever variables might act as determinants in the development of students in relation to their academic environment. In this way, a teaching team would be able to detect the problem and intervene before it fully presents itself, by applying measures that eliminate possible deficiencies in student adaptation to the academic context (Grañeras et al., 2011; Prior et al., 2011; Geary and VanMarie, 2016). For this purpose, these objective indicators of learning difficulties should be detected during the first years of schooling (Alloway and Alloway, 2010; Navarro-Soria and González-Gómez, 2010; Murray and Harrison, 2011; Schmitt et al., 2012; Peng and Fuchs, 2016), since it would facilitate an individualized intervention and the initiation of those psycho-pedagogical resources, which currently act on the problem from a welfare perspective (Rogowsky et al., 2013), rather than from a preventive perspective (Bergman et al., 2011; Raver et al., 2011; Blair and Raver, 2014; Blair et al., 2016; Di Lieto et al., 2017).

From the documentation process involved in the present study, it can be affirmed that intelligence and aptitude are the most researched cognitive phenomena in the body of literature pertaining to the field of psychology. Notwithstanding, among the most prominent authors, there is no clear agreement regarding what intelligent behavior is, whether its nature is inherited or learned, whether it has a static or dynamic capacity, how we can better evaluate it r, or in the case of a psychoeducational intervention if this would allow us to enhance its development (Nisbett et al., 2012; Sternberg, 2012; Richardson and Norgate, 2014; Pietschnig and Voracek, 2015). However, a greater consensus is reached when regarding that intelligence and the differential abilities that compose it are the most influential variables when predicting academic success (Sternberg and Kaufman, 1998; Gagné and Père, 2001; Kuncel et al., 2004; Fergusson et al., 2005; Strenze, 2007; Kytala and Lehto, 2008; Taub et al., 2008). In fact, both classical and current research emphasize that Verbal Aptitude is the best predictor of academic performance, followed by Numerical Aptitude and Reasoning Aptitude and lastly, Spatial Orientation (Smith, 1964; Maccoby and Jacklin, 1974; Burnet and Lane, 1980; Cooper and Reagan, 1982; Kenney-Benson et al., 2006; Prior et al., 2011; Hawes et al., 2015; Duffy et al., 2020). This aptitude hierarchy is modified when it comes to predicting areas of academic performance that require a specific knowledge such as Mathematics. In this case, Numerical Aptitude takes first place in the hierarchy, displacing Verbal Aptitude, which occupies a second position (Marrero and Espino, 1988; Spinath et al., 2006). Similarly, when the area to be predicted is closely related to language management, Numerical Aptitude may be displaced to third position, with Logical Reasoning Aptitude taking second place. The conclusions reached by these previous investigations and other more current investigations, is that an important part of the learning difficulties in the academic context find their etiology in the cognitive aptitudes that are required for the development of traditional school activities (Bennett et al., 2000; Pérez et al., 2005; Miñano and Castejón, 2008; Robles and Vázquez, 2014; Navarro-Soria, 2016; Navarro-Soria et al., 2020).

Based on the above description, we consider it important to study which psychometric tools present, at an early age of the evaluated student, an adequate predictive capacity for academic performance, since the results of the implementation of these tests could be the trigger for a whole series of pedagogical support measures that anticipate school failure. The main interest of this research lies in analyzing the predictive capacity of one of the most widespread tests at the school level, within the bounds of the Spanish territory. Hence, the main objective of this research was to verify that cognitive skills measured in Early Childhood Education, using the Spanish Primary School Aptitude Test (hereinafter termed AEI, Spanish acronym of Aptitudes en Educación Infantil) Battery, have a greater predictive capacity for academic performance throughout Primary Education. The specific aims of the present study were: (a) to verify which cognitive aptitudes have greater predictive capacity with respect to the non-repetition of school years by the student at the end of Primary Education; (b) to evaluate whether the use of oral language (Verbal Aptitude) presents predictive capacity of academic success in the instrumental subjects (Mathematics and Language); (c) to determine whether the knowledge of basic numerical concepts (Numerical Aptitude) presents a predictive power of academic success in the instrumental subjects; (d) to verify whether Spatial Orientation and Reading and Writing Maturity have a predictive power of academic success in the instrumental subjects; (e) to determine whether General Intelligence presents a predictive power of academic success for the instrumental subjects.

In relation to the proposed objectives, our research hypotheses are: (Ha) Verbal Aptitude and Numeric Aptitude will have the greatest predictive power for the non-repetition of the course by the student at the end of Primary Education (a student repeats if he fails math and language at the end of the course); (Hb) Verbal Aptitude, which determines the correct use of language and its different dimensions, will be the aptitude with greater predictive capacity of future academic success for the Language discipline; (Hc) Numerical Aptitudes will be decisive in explaining academic success in Mathematics as in Language; (Hd) Reading-Writer Maturity and Spatial Orientation will present a predictive power of low academic achievement; (He) General Intelligence, contrary to what many studies in the literature claim and despite having some predictive power, will not be among the most powerful aptitudes when it comes to predicting both non-repetition of a school year throughout Primary Education, as with the academic success for the instrumental subjects.

## Materials and Methods

### Participants

In the present study, the sample consisted of the total number of students enrolled in Early Childhood Education (5 years of age) from the four public schools of the province of Alicante (Spain), with a sum total of 631 students. In undertaking a longitudinal study of the sample during the complete period of Primary Education, at the end of the follow-up those students who did not have continuity in their schooling in the municipality and who were unable to complete their follow-up in terms of their evolution have been excluded. Consequently, after the follow-up the total sample was reduced to 512 students. Furthermore, the inclusion criterion used was that students should maintain their schooling in the same school, from Infant Education starting at 5 years of age until completion of the Primary Education stage (12 years of age).

The experimental group of students consisted of 232 males (45.4%) and 280 females (54.6%). The socioeconomic level of the families was distributed among families with income in categories of middle-high with 92 students (18%), middle with 220 students (43%), middle-low with 122 students (24%), and low with 78 students (15%). With regard to the academic training of the families of the students that made up the sample, 22% (113) had undergone university studies, 28% (144) had chosen a vocational training, 41% (209) had finished secondary school and 9% (46) did not have neither an academic background nor qualified training had an academic background or some qualified training.

Before initiating student evaluation and monitoring in this research, the procedure was presented before the District Education Board, which is the highest decision-making body of an educational center in Spain. The District Education Board, related to the four target schools from where the sample was to be drawn, approved the research procedure for those schools. In addition, the psychometric evaluation and the corresponding analysis of the results of the evaluation were reported to all families through mail. In the letter that the families received, families were given the option for their children to not participate in the investigation. None of the families of the total sample exempted their children from participating in the evaluation and subsequent follow-up. This research was conducted in accordance with the 1964 Declaration of Helsinki and its later amendments. Finally, approval was requested from the Ethics Committee of the University of Alicante, which provides and approves the methodology used, and the approval was assigned the file number UA-2018-03-08.

### Instruments

The Spanish Primary School Aptitude Test (AEI) was designed with the objective of evaluating aptitudes that are essential for school learning (Numerical Aptitude, Verbal Aptitude, Spatial Orientation, Auditory Memory, Visomotricity, and Reading and Writing Maturity). One of its great potentials lies in the fact that it allows to measure transcendental cognitive aptitudes for the academic field, long before students have started reading and writing. The Verbal Aptitude and Numerical Aptitude Scales are each composed of 21 items, while the Spatial Orientation Scale consists of 9 items. In these three scales, the student must mark with a cross the image that has the best correspondence to the instruction provided by the evaluator (Lakin and Gambrell, 2012; Lohman and Gambrell, 2012). The Auditory Memory Scale consists of the student listening to the names of a series of objects and then being presented with a template that has a set of images, among which are the aforementioned objects that must be marked with a cross. The Visomotricity Scale consists of three sub-tests, which require the student to draw different figures. All of the scales are scored in relation to the successes or errors of each of the items that compose it. This direct score can be transformed into a percentile score. From the results of the different scales, a Total Score Index and a Reading and Writing Maturity Index are obtained.

This is one of the most widespread tests within the Spanish territory. At a professional level, school counselors use this test to verify the maturity level of the student with respect to acquiring reading and writing skills during the first year of Primary Education, with the scale having a sample of more than 12.000 subjects nationwide. The purpose of the AEI tool is to provide primary school teachers with basic information on the maturity level of each student in order to adapt the rhythms and contents of the program to the real needs of each student in different academic areas. However, from the perspective of the present study, the intention of the AEI tool is the early detection of students with suspected deficiencies in their aptitudes which should be the object of specific work that is aimed at maturation (Navarro-Soria and González-Gómez, 2010).

To check internal consistency, the AEI Battery was subjected to the Cronbach alpha coefficient, obtaining a total score of 0.90 for the data collected from the control sample while this same coefficient was 0.87 for our study sample. In addition, reliability studies were carried out on the AEI Battery using the split-half method, with a result of 0.68 for the sample of 4 year olds and 0.90 for the group of 5 year olds.

Another important psychometric element is validity, in this case the relationship between the different tests of the battery. This data will indicate whether the variations in subject’s performances are due to differences in aptitude. The data from the correlations can be observed in Table 1, as well as the results obtained by the 4 and 5 year olds through which the scale has been elaborated.

**TABLE 1:** AEI Battery reliability (De la Cruz, 1999).

	Verbal	Quantitative	Spatial O.	Memory	Viso. A + B + C	Total	Reading and writing mat.
Verbal aptitude		0.65	0.23	0.28	0.31	0.65	0.59
Numerical aptitude	0.41		0.34	0.27	0.47	0.73	0.61
Spatial orientation	0.24	0.39		-0.10	0.46	0.76	0.80
Auditive memory	0.23	0.23	0.15		0.17	0.31	0.29
Visomotricity	0.18	0.36	0.38	0.14		0.77	0.78
Total	0.53	0.70	0.79	0.39	0.71		0.98
Reading and writing maturity	0.49	0.54	0.82	0.39	0.72	0.97	

### Procedure

In order to verify that cognitive skills, as measured by the AEI Battery, are more influential for academic development, the team of school psychologists conducted an aptitude assessment at the end of the final year of Early Childhood Education (5 years of age), with the subsequent follow-up, at the level of achievement of curricular objectives for the reference course (passed/failed) and promotion to the next level (not failing the two instrumental subjects), throughout 6 years of schooling, collecting information annually on the performance of the sample during their development in Primary Education. The follow-up consisted of annual meetings in which information was collected on regular school attendance, family involvement, changes in family structure, and diagnosis of learning difficulties/disorders. In case of any of these circumstances, and after assessing that this situation could be affecting the performance of the student, the research team decided upon the continuity of the participant in the sample. Therefore, the exclusion criteria for the groups in which the follow-up was carried out were: absenteeism, diagnosis of a clinical disorder and/or family breakdown or negligence.

The evaluation of the students that made up the sample was undertaken during the last quarter of the 5-year period of Early Childhood Education, at which time the AEI psychometric test was implemented (De la Cruz, 1999). The application of this test is collective hence it was carried out by two researchers, one providing instructions for its realization (“Cross the drawing of the open hand, cross the one with hair, cross the drawing of food”), while the other researcher supported those students who presented some difficulties in understanding the test. Its implementation takes approximately 60 min. The student does not require reading and writing skills, since the answering consists of the recognition of images following oral instructions that the evaluator provides to the group during the implementation of the test.

To verify the academic performance of the students, a follow-up of the achievement of curricular objectives (observing if students achieve a pass/fail) in the instrumental subjects (Mathematics and Language) has been undertaken throughout Primary Education, whether the curricular objectives have been passed or not has been used to analyze the differences between students. Taking into account their cognitive abilities (high, medium or low) and based on their school results (pass or fail), the influence of their aptitude differences in their academic performance has been determined.

### Statistical Analysis

To analyze the influence of the predictor variables, logistic regression was used following the Forward Selection procedure based on the Wald statistic. Logistic modeling allows estimation of the probability of an event, a success, or a result occurring, as opposed to not occurring, in the presence of one or more predictors. This probability is estimated by using the odds ratio (OR) statistic. If the OR is greater than one, for each time the event occurs in the absence of the independent variable, it will be given twice if that independent variable is present. On the contrary, if the OR is less than one, the probability that the event occurs in the absence of the independent variable will be greater than if that independent variable were present (De Maris, 2003). To analyze the adjustment of the proposed models, two indicators were taken into account: (a) Nagelkerke’s R^2^ (an adjusted version of the Cox and Snell R^2^ that adjusts the scale of the statistic to cover the full range from 0 to 1), which indicates the percentage of variance explained by the model (Nagelkerke, 1991) and (b) the percentage of correctly classified cases, which allows to determine to what extent the predictor variable is useful for estimating the criterion variable in the proposed model.

## Results

The data permitted the creation of the logistic regression models, which make it possible to effect correct estimations regarding the probability of passing the two instrumental subjects (Language and Mathematics) and not repeating the school year, based on the scores in Cognitive Maturity (Total Index Score) in the different cognitive aptitudes evaluated: Verbal Aptitude (μ = 12.82; *SD* = 3.27), Numerical Aptitude (μ = 13.07; *SD* = 2.98), Spatial Orientation (μ = 13.89; *SD* = 6.38), Auditory Memory (μ = 3.62; *SD* = 1.99), Visomotricity (μ = 16.37; *SD* = 4.56), and Total Score (μ = 59.70; *SD* = 14.41).

Table 1 shows the steps followed by the model in the introduction of explanatory variables that have been significant for the probability of passing the instrumental language subject. For the evaluation using the AEI of a child of 5 years of age, the proposed model allows a correct estimation of 72% of the cases in Verbal Aptitude, 71% in Numerical Aptitude, 74% in Spatial Orientation, 61.5% in Auditory Memory, 68.2% in Visomotricity, 75% in Reading and Writing Maturity, and 75.4% in Total Score.

Nagelkerke’s R^2^ oscillates in the estimation of the adjustment value between0.05 for Auditory Memory and0.40 for both Reading and Writing Maturity and Total Score.

The odds ratio (OR) obtained for the elaborated models of the sample oscillates between 1.11 for Total Score and 1.51 for Numerical Aptitude (Table 2). Thus, during Primary Education the probability that students do not fail the subject of Language, is higher for each incremental point in the result obtained in the following indices: Verbal Aptitude (39%), Numerical Aptitude (47%), Spatial Orientation (20%), Auditory Memory by (23%), Visomotricity (23%), Reading and Writing Maturity (13%), and Total Score (11%), for the entire sample and by entering the variables one by one.

**TABLE 2:** Logistic regression for the predictive probability of each one of the cognitive aptitudes of the AEI Battery and non-failure of the Language instrument.

	Language							
Variable	χ^2^	*R*^2^	*B*	E.T.	Wald	*p*	OR	C.I. 95%
Verbal aptitude	109.73	0.26	0.33	0.03	81.60	<0.001	1.39	1.30–1.50
		Constant −3.99		0.48	67.62	<0.001	0.01	
Numerical aptitude	112.81	0.27	0.39	0.04	80.39	<0.001	1.47	1.35–1.61
		Constant −4.79		0.57	69.52	<0.001	0.00	
Spatial orientation	134.68	0.31	0.18	0.01	101.02	<0.001	1.20	1.16–1.25
		Constant −2.29		0.27	67.19	<0.001	0.10	
Auditive memory	21.36	0.05	0.21	0.04	20.55	<0.001	1.23	1.12–1.35
		Constant −0.47		0.19	6.15	0.01	0.62	
Visomotricity	88.74	0.21	0.21	0.02	70.21	<0.001	1.23	1.17–1.29
		Constant −3.12		0.41	56.36	<0.001	0.04	
Reading and writing maturity	179.57	0.40	0.12	0.01	110.56	<0.001	1.13	1.11–1.16
		Constant −5.71		0.58	95.55	<0.001	0.00	
Total score	181.56	0.40	0.11	0.01	112.34	<0.001	1.11	1.09–1.13
		Constant −6.24		0.62	98.85	<0.001	0.00	

**χ^2^, Chi-Square; R^2^, R^2^ Nagelkerke; B, Coefficient; p, probability; OR, Odds Ratio; C.I., Confidence Interval.**

In Table 3, the introduction of explanatory variables that have been significant for the probability of not failing the instrumental subject of Mathematics is shown. For the evaluation using AEI of a child of 5 years of age, the proposed model allows a correct estimation of 71.8% of cases in Verbal Aptitude, 69.6% in Numerical Aptitude, 72.6% in Spatial Orientation, 62.4% in Auditory Memory, 69.2% in Visomotricity, 76% in Reading and Writing Maturity, and 75.8% in Total Score.

**TABLE 3:** Logistic regression for the predictive probability of each one of the cognitive aptitudes of the AEI Battery and non-failure of the Mathematical instrument.

	Mathematics							
Variable	χ^2^	*R*^2^	*B*	E.T.	Wald	*p*	OR	C.I. 95%
Verbal aptitude	109.23	0.26	0.33	0.03	81.25	<0.001	1.39	1.30–1.50
		Constant −4.01		0.48	68.03	<0.001	0.01	
Numerical aptitude	124.13	0.29	0.41	0.04	85.80	<0.001	1.52	1.39–1.66
		Constant −5.17		0.59	75.51	<0.001	-5.17	
Spatial orientation	143.63	0.33	0.19	0.01	105.10	<0.001	1.21	1.17–1.26
		Constant −2.44		0.28	72.06	<0.001	0.08	
Auditive memory	16.45	0.04	0.18	0.04	15.97	<0.001	1.20	1.10–1.32
		Constant −0.40		0.18	4.50	0.03	0.67	
Visomotricity	85.60	0.21	0.20	0.02	68.26	<0.001	1.22	1.17–1.29
		Constant −3.07		0.41	55.30	<0.001	0.46	
Reading and writing maturity	180.63	0.40	0.13	0.01	110.78	<0.001	1.13	1.11–1.16
		Constant −5.78		0.58	96.46	<0.001	0.00	
Total score	183.62	0.41	0.11	0.01	112.89	<0.001	1.11	1.09–1.14
		Constant −6.34		0.63	100.04	<0.001	0.00	

**χ^2^, Chi-Square; R^2^, R^2^ Nagelkerke; B, Coefficient; p, probability; OR, Odds Ratio; C.I., Confidence Interval.**

Nagelkerke’s R^2^ oscillates in the estimate of the adjustment value between0.04 for Auditory Memory and 0.41 for Total Score.

The odds ratio (OR) that was obtained for the elaborated models of the sample oscillates between 1.11 for Total Score and 1.52 for Numerical Aptitude. Thus, the probability that students do not fail the instrumental subject of Mathematics during any of the Primary Education courses, is greater for each incremental point in the result obtained in the following indices: Verbal Skills (39%), Numerical Aptitude (52%), Spatial Orientation (21%), in Auditory Memory (20%), Visomotricity (22%), Reader and Writing Maturity (13%), and Total Score (11%), for the entire sample and by entering the variables one by one.

In Table 4 are the steps followed by the model in the introduction of explanatory variables that have been significant for the probability of not repeating a school year. For the evaluation with AEI of a child of 5 years of age, the proposed model allows a correct estimation of 89.2% of the cases in Verbal Aptitude, 88% in Numerical Aptitude, 88.4% in Spatial Orientation, 87.5% in Auditory Memory, 87.4% in Visomotricity, 90% in Reading and Writer Maturity, and 90.6% in Total Score.

**TABLE 4:** Logistic regression for the predictive probability of each one of the cognitive aptitudes of the AEI Battery and non-repetition of a school year.

	Repetition							
Variable	χ^2^	*R*^2^	*B*	E.T.	Wald	*p*	OR	C.I. 95%
Verbal aptitude	71.73	0.25	0.35	0.04	57.86	<0.001	1.42	1.29–1.55
		Constant −2.11		0.56	17.48	<0.001	0.12	
Numerical aptitude	76.54	0.26	0.41	0.05	55.96	<0.001	1.51	1.35–1.68
		Constant −3.00		0.63	22.52	<0.001	0.05	
Spatial orientation	94.05	0.32	0.22	0.02	68.02	<0.001	1.24	1.18–1.31
		Constant −0.40		0.26	2.42	0.11	0.66	
Auditive memory	18.37	0.06	0.28	0.06	17.72	<0.001	1.33	1.16–1.53
		Constant 1.01		0.23	18.81	<0.001	2.76	
Visomotricity	66.57	0.23	0.26	0.03	52.18	<0.001	1.30	1.21–1.39
		Constant −1.89		0.49	14.38	<0.001	0.15	
Reading and writing maturity	126.83	0.42	0.13	0.01	77.04	<0.001	1.14	1.11–1.82
		Constant −3.63		0.58	38.04	<0.001	0.02	
Total score	124.38	0.41	0.11	0.01	75.43	<0.001	1.12	1.09–1.15
		Constant −4.23		0.66	41.07	<0.001	0.01	

**χ^2^, Chi-Square; R^2^, R^2^ Nagelkerke; B, Coefficient; p, probability; OR, Odds Ratio; C.I., Confidence Interval.**

Nagelkerke’s R^2^ oscillates in the estimation of the adjustment value between 0.06 for Auditory Memory and 0.42 for Reading and Writing Maturity.

The odds ratio (OR) that was obtained for the elaborated models of the sample oscillates between 1.12 for Total Score and 1.51 for Numerical Aptitude. Thus, the probability that students do not repeat a school year during Primary Education is greater for each incremental point in the result obtained in the following indices: Verbal Aptitudes (42%), in Numerical Aptitudes (51%), in Spatial Orientation a (24%), in Auditory Memory (33%), in Visomotricity (30%), in Reading and Writing Maturity (14%) and in Total Score (12%), for the whole sample and introducing the variables one by one.

## Discussion

From the analysis of the results, we conclude that Verbal and Numerical Aptitudes are the variables with greater predictive capacity respect to the development of the different competences academic and probability of repeating a course. Thus, the results confirm that the AEI tool has a good predictive capacity for Early Childhood Education with respect to the appearance of difficulties in overcoming the curricular objectives during Primary Education, thus confirming the hypothesis H_*a*_.

When evaluating student aptitude development using the AEI battery prior to the start of Primary Education, it was noted that for each incremental point in the results for Verbal and Numerical Aptitudes, the probability that the student does not repeat an academic year oscillates between 42 and 51%, respectively. These data reinforce the results of previous research that also conclude that Verbal and Numerical Aptitudes are valid predictors of learning difficulties (Burnet and Lane, 1980; Cooper and Reagan, 1982; Bennett et al., 2000; Pérez et al., 2005; Robles and Vázquez, 2014). This conclusion underlines the importance of these skills being acquired through specific training to enhance their development. Therefore, training in Verbal and Numerical Aptitudes is considered an adequate academic response which favors acquisition by students of the curricular objectives that are proposed for the different disciplines in the first cycles of Primary Education.

In the same line, when the results are analyzed with a focus on which aptitudes present a greater predictive capacity, with a possible failure of the instrumental subjects throughout Primary Education, it was observed that in the AEI Battery and for the instrumental subject of Language, it is Numerical Aptitude which, with a slightly higher result (72.6%), is ahead of Verbal Aptitude (69.6%) at the level of predictive capacity, confirming the hypotheses H_*b*_ and H_*c*_. These results, which initially could seem inconsistent, are justified if one considers that in the AEI test, Numerical Aptitude is measured by using the knowledge of verbal concepts that are associated with learning Mathematics (for example: more/less, bigger-number/smaller-number, same/different, superior/inferior, first/last, equal/not equal, etc.). This is a numerical vocabulary that is less used in everyday conversations with children at this age, but it would measure a deeper knowledge of Language. On the one hand, different authors in the literature have highlighted Mathematical Competence during the first school years as a powerful predictor of academic success, not only for the discipline itself but also for other domains such as Language (Aubrey et al., 2006; Duncan et al., 2007; Jordan et al., 2010; Geary et al., 2013; Martin et al., 2014; Park et al., 2016).

On the other hand, indices such as Spatial Orientation, Reading and Writing Maturity and Visomotricity do not stand out as predictors of academic success in the instrumental subject of Language, which contrasts with the results obtained in other investigations where these skills are understood to be crucial in the acquisition of Reading and Writing skills (Frostig et al., 1964; Smith, 1964; Mlodnosky, 1968; Bender, 1977; Koppitz, 1980; Valett, 1989; Spitz, 2009), thus confirming the hypothesis H_*d*_. It is possible that these data could present a more significant difference in relation to the other indices if, instead of making the estimate by taking into account the whole of Primary Education, the results would be isolated at the academic level during the First Cycle of Primary Education in the subject of Language. In that, the First Cycle of Primary Education is a particular period when the acquisition of Reading and Writing skills is a priority objective and they are academic competences that require adequate maturity at the level of Spatial Orientation, Reading and Writing Maturity, and Visomotricity. In the same way, the Spatial Orientation Index, the Reading and Writing Maturity Index and the Visomotricity Index do not present any significant relevance at the level of predictive capacity of academic success in Mathematics, which also contrasts with recent research, for which the results highlight that the Visuospatial Skills Index, measured between the ages 5 and 6 years, is a powerful predictor of success in the field of mathematics (Cheng and Mix, 2014; Hawes et al., 2015; Pitchford et al., 2016; Cornu et al., 2017, 2018).

A further notable result, that coincides with various investigations in the body of literature, is that General Intelligence is not a determining variable in predicting school success (Edel, 2003; Laidra et al., 2006; Deary et al., 2007; Watkins et al., 2007; Miñano and Castejón, 2008). While it is true that this statement is controversial and has many defenders as well as detractors, it is not difficult to find a diversity of research which determines that General Intelligence can be considered as one of the best predictors of academic success (Deary et al., 2007; Kaufman et al., 2012; Roth et al., 2015; Schult and Sparfeldt, 2016; Gygi et al., 2017). However, for our sample and with the aptitude being measured using the AEI tool, the fact of having higher scores in this particular index has not been associated with greater possibilities of non-repetition of school years. Therefore, although General Intelligence has some influence over success with respect to achieving the curricular objectives of the reference course, it is not the most determining factor among the measured aptitudes, thus confirming the hypothesis H_*e*_.

Finally, if the results for the aptitudes that are measured with the AEI tool and the instrumental subject of Mathematics are observed and analyzed, the Numerical Aptitude Index stands out as the best predictor of possible failure, over and above the rest of the aptitudes/skills. This fact is indeed justified. In that, if a student in the First Year of Primary Education does not understand the concepts that are used to explain the numerical operations, they will find great difficulties in correctly executing exercises in Mathematics, due to the great level of abstraction required by cognitive processes associated with this type of activity at this educational level. These results are in agreement with those obtained in recent investigations (Matthews et al., 2015; Ye et al., 2016; Harvey and Miller, 2017), whose conclusions highlight the importance of the development of the language of Mathematics in Early Childhood Education for the adequate acquisition of Mathematics in later stages of the educational process.

In terms of study limitations, it is noteworthy that for future investigations, the sample of both subjects and provinces in which information was collected should be expanded, and this is among the most important limitations of this study. In addition, it would be advisable to not limit the study to a single evaluation instrument, but to include different psychometric tests that allow the collection of data from other variables beyond cognitive aptitudes, in order to be able to assert in a more substantial manner what academic successes and failures can be attributed to.

## Conclusion

The most relevant conclusion of this study is the finding that a psychometric tool such as AEI presents an adequate capacity to predict academic success or failure in the instrumental subjects of Mathematics and Language. In that, these two instrumental subjects provide the basis for the remainder of the required academic knowledge. This predictive capacity is available throughout 6 years of schooling before any difficulties are evidenced. One can assume that this is an ample period of time in which to be able to promote preventive actions that could reduce the probability of academic failure.

Based on the obtained results, we propose that the AEI tool can be considered effective in anticipating learning difficulties when it is included in an early detection program that aims to implement pedagogical strategies which favor an adequate maturity at the student aptitude level, thus avoiding any future academic failure. The AEI tool should be linked to the development of models of psychopedagogical intervention (Prior et al., 2011; Raver et al., 2011; Geary and VanMarie, 2016) which are aimed at reducing learning difficulties in the First Cycles of Primary Education. At that stage, if student difficulties go unnoticed or they do not receive the necessary focus or importance, the first deficiencies will affect student learning capacity, which in many cases will then accumulate in each following school year. In fact, recent research (Serpell and Esposito, 2016) highlights the importance of this type of strategy being transferred from government institutions through relevant legislation to educational programs that are aimed at the early prevention of learning difficulties, in order to reduce the worrying current rates of school failure in Spain.

As a final conclusion and in agreement with other research where by data were collected during Primary Education and Secondary Education (Duncan et al., 2007; Murray and Harrison, 2011; Serpell and Esposito, 2016; Harvey and Miller, 2017), we wish to emphasize that early academic performance is a robust predictor of later academic ability. Children with greater knowledge and understanding of Mathematics and Language concepts at the beginning of compulsory schooling achieve higher academic levels in later education than their less-prepared peers. Regarding the relevance of this research and the data obtained from it, the Spanish Primary School Aptitude Test (AEI) Battery is an adequate prediction tool at an early age which provides technical arguments and objectives for initiating reinforcement measures at an initial level of schooling, in order to avoid later learning difficulties that may arise.

## Data Availability Statement

The raw data supporting the conclusions of this article will be made available by the authors, without undue reservation.

## Ethics Statement

The studies involving human participants were reviewed and approved by the Ethics Committee of the University of Alicante, which provides and approves the methodology used, and the approval was assigned the file number UA-2018-03-08. Written informed consent to participate in this study was provided by the participants’ legal guardian/next of kin.

## Author Contributions

IN-S contributed to the project idea, article concept, and design, as well as planning the timeline, substantially involved in the data, material, and article acquisition, mainly responsible for drafting, writing, and revising the review article, and responsible for selection and final approval of the scholarly publication. JÁ-T participated in drafting the work and revising it critically with respect to important intellectual content in all its phases. LG-A participated in drafting the work and revising it critically with respect to important intellectual content in all its phases. RL-C provided substantial help with the concept and design, contributed substantially to the project by drafting and revising the review article, and being responsible for the final approval of the scholarly publication. All authors contributed to the article and approved the submitted version.

## Conflict of Interest

The authors declare that the research was conducted in the absence of any commercial or financial relationships that could be construed as a potential conflict of interest.

## Abbreviations

AEI: Spanish Primary School Aptitude Test
χ ^2^: Chi-Square
*R*^2^: *R*^2^ Nagelkerke
B: Coefficient
p: probability
OR: Odds Ratio
C.I.: Confidence Interval.

## References

[B1] Alloway T. Alloway R. (2010). Investigating the predictive roles of working memory and IQ in academic attainment. *J. Exp. Child Psychl.* 106 20–29. 10.1016/j.jecp.2009.11.003 20018296

[B2] Alloway T. Passolunghi M. (2011). The relationship between working memory, IQ, and mathematical skills in children. *Learn. Individ. Differ.* 21 133–137. 10.1016/j.lindif.2010.09.013

[B3] Aubrey C. Godfrey R. Dahl S. (2006). Early mathematics development and later achievement: further evidence. *Math. Educ. Res. J.* 18 27–46. 10.1007/BF03217428

[B4] Bender L. (1977). *El Test Guestaltico Visomotor: Usos y Aplicaciones Clínicas*. México: Paidós.

[B5] Bennett G. K. Seashore H. G. Wesman A. G. (2000). *Differential Aptitude Tests (DAT-5).Manual.* Madrid: TEA Ediciones.

[B6] Bergman S. Söderqvist S. Bryde S. Thorell L. B. Humphreys K. Klingberg T. (2011). Gains in fluid intelligence after training non-verbal reasoning in 4-year-old children: a controlled, randomized study. *Dev. Sci.* 14 591–601. 10.1111/j.1467-7687.2010.01022.x 21477197

[B7] Blair C. McKinnon R. D. The Family Life Project Investigators. (2016). Moderating effects of executive functions and the teacher–child relationship on the development of mathematics ability in kindergarten. *Learn. Instruct.* 41 85–93. 10.1016/j.learninstruc.2015.10.001 28154471 PMC5283384

[B8] Blair C. Raver C. (2014). Closing the achievement gap through modification of neurocognitive and neuroendocrine function: results from a cluster randomized controlled trial of an innovative approach to the education of children in Kindergarten. *PLoS One* 9:e112393. 10.1371/journal.pone.0112393 25389751 PMC4229187

[B9] Bonti E. Tzouriadou M. (2015). Low visual-perceptive ability cognitive profile places urban Greek kindergarten children “al risk” for dyslexic learning difficulties. *Educ. Urb. Soc.* 47 646–668. 10.1177/0013124513499184

[B10] Burnet S. Lane C. M. (1980). Effects of academic instruction of spational visualization. *Intelligence* 4 233–247. 10.1016/0160-2896(80)90021-5

[B11] Carlson A. Rowe E. Curby T. (2013). Disentangling fine motor skills’ relation to academic achievement: the relative contributions of visual-spatial integration and visual-motor coordination. *J. Genet. Psychol.* 174 514–533. 10.1080/00221325.2012.717122 24303571

[B12] Cassidy S. Roche B. Colbert D. Stewart I. Grey I. M. (2016). A realional fram skills training intervention to increase general intelligence and scholastic aptitude. *Learn. Individ. Differ.* 47 222–235. 10.1016/j.lindif.2016.03.001

[B13] Cerda G. Pérez C. Navarro J. I. Aguilar M. Casas J. A. Aragón E. (2015). Explanatory model of emotional-cognitive variables in school mathematics performance: a longitudinal study in primary school. *Front. Psychol.* 6:1363. 10.3389/fpsyg.2015.01363 26441739 PMC4561756

[B14] Cheng Y. L. Mix K. S. (2014). Spatial training improves children’s mathematics ability. *J. Cogn. Dev.* 15 2–11. 10.1080/15248372.2012.725186

[B15] Cooper L. Reagan D. (1982). “Attention, perception and intelligence,” in *Handbook of human Intelligence*, ed. Sternberg R. J. (Cambridge: Cambridge University Press), 123–170.

[B16] Córdoba L. García V. Luengo L. Vizuete M. Feu S. (2011). Sociocultural determinants. Their relationship with the academic performance of students in Compulsory Secondary Education. *Rev. Investigac. Educ. (RIE)* 29 83–96.

[B17] Cornu V. Schiltz C. Martin R. Hornung C. (2018). Visuo-spatial abilities are key for Young children’s verbal number skills. *J. Exp. Child Psychol.* 166 604–620. 10.1016/j.jecp.2017.09.006 29107883

[B18] Cornu V. Schiltz C. Pazouki T. Martin R. (2017). Training early visuo-spatial abilities: a controlled classroom-based intervention study. *Appl. Dev. Sci.* 23 1–21. 10.1080/10888691.2016.1276835

[B19] De la Cruz M. V. (1999). *Aptitudes in Infantile Education (AEI): Preschool at the Age of 2 Years.* Madrid: TEA Ediciones.

[B20] De Maris A. (2003). “Logistic regression,” in *Research Methods in Psychology*, eds Schinka J. A. Velicer W. F. (Hoboken, NJ: John Wiley and Sons), 509–532.

[B21] Deary I. J. Strand S. Smith P. Fernandes C. (2007). Intelligence and educational achievement. *Intelligence* 35 13–21. 10.1016/j.intell.2006.02.001

[B22] Di Lieto M. Inguaggiato E. Castro E. Cecchi F. Cioni G. Dell’Omo M. (2017). Educational robotics intervention on executive functions in preschool children: a pilot study. *Comput. Hum. Behav.* 71 16–23. 10.1016/j.chb.2017.01.018

[B23] Duckworth A. Yeager D. (2015). Measurement matters. Assessing personal qualities other than cognitive ability for educational purposes. *Educ. Res.* 4 237–251. 10.3102/0013189X15584327 27134288 PMC4849415

[B24] Duffy G. Sorby S. Bowe B. (2020). An investigation of the role of spatial ability in representing and solving word problems among engineering students. *J. Eng. Educ.* 109 424–442. 10.1002/jee.20349

[B25] Duncan G. Dowsett C. Claessens A. Magnuson K. Huston A. Klebanov P. (2007). School readiness and later achievement. *Dev. Psychol.* 43 1428–1446. 10.3389/fpsyg.2017.00132 18020822

[B26] Edel R. (2003). Factores asociados al rendimiento académico. *REICE. Revista Electrónica Iberoamericana sobre Calidad, Eficacia y Cambio en la Educación*. 20, 112–121.

[B27] Fergusson D. Horwood J. Ridder E. (2005). Show me the child at seven II: childhood intelligence and later outomes in adolescence and young adulthood. *J. Child Psychol. Psychiatry* 46 850–858. 10.1111/j.1469-7610.2005.01472.x 16033633

[B28] Frostig M. Lefever D. W. Whittlesey R. B. (1964). *The Marianne Frostig Developmental Test of Visual Perception.* Palo Alto, CA: Consulting Psychologists Press.

[B29] Gagné F. Père F. (2001). When IQ is controlled, does motivation still predict achievement? *Intelligence* 30 71–100. 10.1016/S0160-2896(01)00068-X

[B30] García-Fernández J. Martínez-Monteagudo M. Inglés C. (2013). How is anxiety at school related to academic performance? *Rev. Iberoam. Psicol. Salud* 4 63–76.

[B31] Garon-Carrier G. Boivin M. Guay F. Kovas Y. Dionne G. Lemelin J. P. (2016). Intrinsic motivation and achievement in mathematics in elementary school : a longitudinal investigation of thei association. *Child Dev.* 87 165–175. 10.1111/cdev.12458 26548456

[B32] Garon-Carrier G. Boivin M. Lemelin J. P. Kovas Y. Parent S. Séguin J. R. (2018). Early developmental trajectories of number knowledge and math achievement from 4 to 10 years: low-persistent profile and early-life predictors. *J. Sch. Psychol.* 68 84–98. 10.1016/j.jsp.2018.02.004 29861033

[B33] Gay G. (2010). *Culturally Responsive Teaching: Theory, Research, and Practice*, 2nd Edn New York, NY: Teachers College Press.

[B34] Geary D. C. Hoard M. K. Nugent L. Bailey D. H. (2013). Adolescents’ functional numeracy is predicted by their school entry number system knowledge. *PLoS One* 8:e54651. 10.1371/journal.pone.0054651 23382934 PMC3559782

[B35] Geary D. C. VanMarie K. (2016). Young children’s core symbolic and nonsymbolic quantitative knowledge in the prediction of later mathematics achievement. *Dev. Psychol.* 52 2130–2144. 10.1037/dev0000214 27736101

[B36] Grañeras M. Díaz-Caneja P. Gil N. (2011). *Success Activation in European Schools.* Madrid: Secretaría General Técnica del Ministerio de Educación.

[B37] Grivins M. (2013). Pupil Grouping. Education agent interaction influence on education results. *REMEI Multidisc. J. Educ. Res.* 2 147–172.

[B38] Gygi J. T. Hagmann-von P. Schweizer F. Grob A. (2017). The predictive validity of four intelligence tests for school grades: a small sample longitudinal study. *Front. Psychol.* 8:375. 10.3389/FPSYG.2017.00375 28348543 PMC5346574

[B39] Harvey H. Miller G. (2017). Executive function skills, early mathematics, and vocabulary in head start preeschool children. *Early Educ. Dev.* 28 290–307. 10.1080/10409289.2016.1218728

[B40] Hawes Z. Moss J. Caswell B. Poliszczuk D. (2015). Effects of mental rotation training on children’s spatial and mathematics performance: a randomized controlled study. *Trends Neurosci. Educ.* 4 60–68. 10.1016/j.tine.2015.05.001

[B41] Hill O. Serpell Z. Faison M. (2016). The efficacy of the LearningRx cognitive training program: modality and transfer effects. *J. Exp. Educ.* 84 600–620. 10.1111/j.1559-1816.2002.tb01421.x

[B42] Jordan N. C. Glutting J. Ramineni C. (2010). The importance of number sense to mathematics achievement in first and third grades. *Learn. Individ. Differ.* 20 82–88. 10.1016/j.lindif.2009.07.004 20401327 PMC2855153

[B43] Kaufman K. A. Reynolds M. R. Liu X. Kaufman A. S. McGrew K. S. (2012). Are cognitive g and academic achievement g on and the same g? *Intelligence* 40 123–138. 10.1016/j.intell.2012.01.009

[B44] Kenney-Benson G. Pomerantz E. Ryan A. Patrik H. (2006). Sex differences in math performance: the role of children’s approach to schoolwork. *Dev. Psychol.* 42 11–26. 10.1037/0012-1649.42.1.11 16420115

[B45] Kern M. L. Friedman H. S. (2009). Early educational milestones as predictors of lifelong academic achievement, midlife adjustment and longevity. *J. Appl. Dev. Psychol.* 30 419–430. 10.1016/j.appdev.2008.12.025 19626128 PMC2713445

[B46] Koppitz E. (1980). *El Test Gestaltico Visomotor Para Niños*. Buenos Aires: Guadalupe.

[B47] Kuncel N. R. Hezlett S. A. Ones D. S. (2004). Academicperformance, career potential, creativity, and job performance:can one construct predict them all*?*. *J. Pers. Soc. Psychol.* 86 148–161. 10.1037/0022-3514.86.1.148 14717633

[B48] Kytala M. Lehto J. E. (2008). Some factors underlyingmathematical performance: the role of visuospatial working memory and non-verbal intelligence. *Eur. J. Psychol. Educ.* 23 77–94. 10.1007/bf03173141

[B49] Laidra K. Pullman H. Allik J. (2006). Personality and intelligence as predictors of academic achievement: a cross-sectional study from elementary to secondary school. *Pers. Individ. Differ.* 42 441–451. 10.1016/j.paid.2006.08.001

[B50] Lakin J. M. Gambrell J. L. (2012). Distinguishing verbal, quantitative, and figural facets of fluid intelligence in young students. *Intelligence* 40 560–570. 10.1016/j.intell.2012.07.005

[B51] Lohman D. F. Gambrell J. (2012). Using nnverbal test to hel identify academically talented children. *J. Psychoeducat. Assess.* 30 25–44. 10.1177/0734282911428194

[B52] Maccoby E. Jacklin C. (1974). *The Psychology of Sex Differences.* Stanford, CA: Stanford University Press.

[B53] Marrero H. Espino O. (1988). Comparative evaluation of the predictive power of aptitude on school grades and objective test scores. *Rev. Educ.* 297 97–112.

[B54] Martin R. B. Cirino P. T. Sharp C. Barnes M. (2014). Number and counting skills in kindergarten as predictors of Grade 1 mathematical skills. *Learn. Individ. Differ.* 34 12–23. 10.1016/j.lindif.2014.05.006 25089081 PMC4116688

[B55] Matthews P. G. Lewis M. R. Hubbard E. M. (2015). Individual differences in nonsymbolic ratio processing predict symbolic math performance. *Psychol. Sci.* 27 191–202. 10.1177/0956797615617799 26710824

[B56] Meneses-Botina W. Morillo-Carlosama S. Navia-Atoy G. Grisales-Grisales M. (2013). Factors affecting school performance in the rural educational institution. The Mercedes (i.e. The Institute of Rural Education) from the perspective of institutional actors. *Plum. Educ.* 11 433–452.

[B57] Miñano P. Castejón J. (2008). Predictive ability of cognitive-motivational variables on academic performance. *Rev. Electrón. Motivac. Emoc.* 28 203–230.

[B58] Miñano P. Castejón J. (2011). Cognitive and motivational variables of academic performance in the Spanish Language and Mathematics: a structural model. *Rev. Psicod.* 16 203–230. 10.1387/RevPsicodidact.930 33433647

[B59] Mlodnosky L. (1968). The Frostig and the bender gestalt as predictors of reading achievement. *Tech. Rep.* 3, 129–139.

[B60] Moliner L. Moliner O. Sales A. (2012). Because we don’t learn much alone: a reciprocal peer tutoring experience in primary education. *Rev. Investigac. Educ.* 30 459–474.

[B61] Murray E. Harrison L. J. (2011). The influence of being ready to learn on children’s early school literacy and numeracy achievement. *Educ. Psychol.* 31 529–545. 10.1080/01443410.2011.573771

[B62] Nagelkerke N. J. (1991). A note on a general definition of the coefficient of determination. *Biometrika* 78 691–692. 10.2307/2337038

[B63] Navarro-Soria I. (2016). *Cognitive, School and Socio-Demographic Predictive Variables of Academic Performance in Primary Education.* Doctoral thesis, Univesidad de Alicante, Alicante. 10.13140/RG.2.2.21077.32482

[B64] Navarro-Soria I. García-Fernández J. M. Inglés C. Real M. (2020). Early detection of learning difficulties using the BADyG-E2r battery durong primary education. *Psicologia* 33:4. 10.1186/s41155-020-00143-y 32382802 PMC7205914

[B65] Navarro-Soria I. González-Gómez C. (2010). “Systematic detection of aptitude deficits as a strategy for preventing academic difficulties,” in *Keys to Research in Innovation and Educational Quality*, eds Roig Vila R. Fiorucci M. (Marfil: Alcoi), 297–309.

[B66] Nisbett R. Aronson J. Blair C. Dickens W. Flynn J. Halpern D. (2012). Intelligence: new findings and theoretical developments. *Am. Psychol.* 67 130–159. 10.1037/a0026699 22233090

[B67] Núñez J. Vallejo G. Rosário P. Tuero E. Valle A. (2014). Student and teacher variables and their context in the prediction of academic performance in Biology: an analysis from a multi-level perspective. *Rev. Psicod.* 19 145–172. 10.1387/RevPsicodidact.7127 33433647

[B68] Park J. Bermudez V. Roberts R. Brannon E. (2016). Non-symbolic approximate arithmetic training improves math performance in preschoolers. *J. Exp. Child Psychol.* 152 278–293. 10.1016/j.jecp.2016.07.011 27596808 PMC5053875

[B69] Peng P. Fuchs D. (2016). A meta-analysis of working memory deficits in children with learning difficulties: is there a difference between verbal domain and numerical domain? *J. Learn. Disabil.* 49 3–20. 10.1177/0022219414521667 24548914

[B70] Pérez E. Cupani M. Ayllón S. (2005). Predictors of academic performance in middle school: skills, self-efficacy and personality traits. *Aval. Psicol.* 4 1–11.

[B71] Phillipson S. Phillipson S. N. (2012). Children’s cognitive ability and their academic achievement: the mediation effects of parental expectations. *Asia Pac. Educ. Rev.* 13 495–508. 10.1007/s12564-011-9198-1

[B72] Pietschnig J. Voracek M. (2015). One century of global IQ gains: a formal meta-analysis of the flynn effect (1909–2013). *Perspect. Psychol. Sci.* 10 282–306. 10.1177/1745691615577701 25987509

[B73] Pitchford N. Papini C. Outhwaite L. Gulliford A. (2016). Fine motor skills predict maths ability better than they predict reading ability in the early primary school years. *Front. Psychol.* 7:783. 10.3389/fpsyg.2016.00783 27303342 PMC4884738

[B74] Prior M. Bavin E. Ong B. (2011). Predictors of school readiness in five- to six- year-old children from an Australian longitudinal community sample. *Educ. Psychol.* 31 3–16. 10.1080/01443410.2010.541048

[B75] Puerta L. (2015). Relationship between cognitive processes and academic performance in high school students. *Psychologia* 9 85–100. 10.21500/19002386.1816

[B76] Raver C. C. Jones S. M. Li-Grining C. Zhai F. Bub K. Pressler E. (2011). CSRP’s impact on low-income preschoolers’ preacademic skills: self-regulation as a mediating mechanism. *Child Dev.* 82 362–378. 10.1111/j.1467-8624.2010.01561.x 21291447 PMC3682645

[B77] Richardson K. Norgate S. (2014). Does IQ measure ability for complex cognition? *Theory Psychol.* 24 795–812. 10.1177/0959354314551163

[B78] Risso A. Peralbo M. Barca A. (2010). Changes in the predictive variables of school performance in Secondary Education. *Psicothema* 22 790–796. 21044515

[B79] Robles C. Vázquez E. (2014). The influence of verbal skills on school success. *Int. J. Dev. Educ. Psychol.* 6 351–362.

[B80] Rogowsky B. A. Papamichalis P. Villa L. Heim S. Tallal P. (2013). Neuroplasticity-based cognitive and linguistic skills training improves reading and writing skills in college students. *Front. Psychol.* 4:137. 10.3389/fpsyg.2013.00137 23533100 PMC3607067

[B81] Rohde T. Thompson L. (2007). Predicting academic achievement with cognitive ability. *Intelligence* 35 83–92. 10.1016/j.intell.2006.05.004

[B82] Roth B. Becker N. Romeyke S. Schäfer S. Domnick F. Spinath F. M. (2015). Intelligence and school grades: a meta-analysis. *Intelligence* 53 118–137. 10.1016/j.intell.2015.09.002

[B83] Schmitt M. B. Pentimonti J. M. Justice L. M. (2012). Teacher-child relationships, behavior regulation, and language gain among at-risk preschoolers. *J. Sch. Psychol.* 50 681–699. 10.1016/j.jsp.2012.04.003 23040762

[B84] Schult J. Sparfeldt J. R. (2016). Do non-g factors of cognitive ability test align with specific academic achievements? a combined bifactor modeling approach. *Intelligence* 59 96–102. 10.1006/jintell.2016.08.004

[B85] Serpell Z. Esposito A. (2016). Development of executive functions. Implications for Educational Policy and practice. *Policy Insigh. Behav. Brain Sci.* 3 203–210. 10.1177/2372732216654718

[B86] Smith I. M. (1964). *Spatial Ability: Its Educational and Social Significance.* San Diego, CA: Knapp.

[B87] Spinath B. Spinath F. M. Harlaar N. Plomin R. (2006). Predicting school achievement from general cognitive ability, self-perceived ability, and intrinsic value. *Intelligence* 34 363–374. 10.1016/j.intell.2005.11.004

[B88] Spitz H. (2009). *The raising of Intelligence: A Selected History of Attemps to Raise Retarded Intelligence.* Mahwah, NJ: Lawrence Erlbaum Associates.

[B89] Sternberg R. (2012). The intelligence of Nations: smart but not wise – A comment on Hunt (2012). *Perspect. Psychol. Sci.* 8 187–189. 10.1177/1745691612443829 26172499

[B90] Sternberg R. Kaufman J. (1998). Human abilities. *Annu. Rev. Psychol.* 49 479–502. 10.1146/annurev.psych.49.1.479 9496630

[B91] Strenze T. (2007). Intelligence and socioeconomic success: a meta-analytic review of longitudinal research. *Intelligence* 35 401–426. 10.1016/j.intell.2006.09.004

[B92] Taub G. Keith T. Floyd R. Mcgrew K. (2008). Effects of general and broad cognitive abilities on mathematicsachievement. *Sch. Psychol. Q.* 23 187–198. 10.1037/1045-3830.23.2.187

[B93] Toll S. M. Van Luit J. E. (2014). Explaining numeracy development in weak performing kindergatners. *J. Exp. Child Psychol.* 124 97–111. 10.1016/j.jecp.2014.02.001 24786672

[B94] Valett R. (1989). *Dislexia*. CEAC: Barcelona.

[B95] Watkins M. Lei P. Canivez G. (2007). Psychometric intelligence and achievement: a cross-lagged panel analysis. *Intelligence* 35 59–68. 10.1016/j.intell.2006.04.005 PMC6526451 31162422

[B96] Ye A. Resnick I. Hansen N. Rodrigues J. Rinne L. Jordan N. (2016). Pathways to fraction learning: numerical abilities mediate the relation between early cognitive competencies and later fraction knowledge. *J. Exp. Child Psychol.* 152 242–263. 10.1016/j.jecp.2016.08.001 27572521

